# Revisiting the musculocutaneous external oblique flap as a versatile alternative in large thoracic wall defects

**DOI:** 10.1186/s40792-019-0708-4

**Published:** 2019-10-22

**Authors:** David Matera, Richard Huynh, Terrance Hanley, Amir B. Behnam

**Affiliations:** 1 Philadelphia College of Osteopathic Medicine, GME office, 4190, City Avenue, Philadelphia, PA 19131 USA; 2 Department of Plastic and Reconstructive Surgery, Philadelphia College of Osteopathic Medicine, 4190 City Avenue, Philadelphia, PA 19131 USA; 3 Department of Surgery, Reading Hospital, Reading, PA USA; 4 Division of Plastic and Reconstructive Surgery, Reading Hospital, Reading, PA USA

**Keywords:** External oblique flap, External oblique musculocutaneous flap, Thoracic wall defect, Chest wall defect, Angiosarcoma

## Abstract

**Background:**

The external oblique myocutaneous flap has been previously described for reconstruction of chest-thoracic wall defects smaller than 400–500 cm^2^. However, it is utilized less often than workhorse flaps such as the omental, pectoralis, rectus abdominis, and latissimus dorsi myocutaneous flaps as many plastic surgeons are not aware that the flap can cover larger areas than previously documented.

**Case presentation:**

We report a 57-year-old female tobacco user who underwent a resection of a grade 3 breast angiosarcoma resulting in a high left chest wall soft tissue defect approximating 900 cm^2^. The patient underwent an external oblique myocutaneous pedicle flap reconstruction of the defect, most notably in anticipation of postoperative adjuvant radiation therapy. No gross flap complications and or patient impairment were noted. Thirteen months status post flap reconstruction, the patient underwent an aortic valve replacement requiring re-elevation of the same flap for exposure. The flap demonstrated excellent viability during the procedure and postoperatively.

**Conclusion:**

The pedicled external oblique myocutaneous flap should be considered when reconstructing larger high chest wall defects when other more common flaps used in chest reconstruction may not be indicated. The external oblique myocutaneous flap is an excellent tool in the armamentarium of any reconstructive surgeon; it is a straightforward and versatile flap that can be safely and reliably used in durable reconstruction of defects of the chest wall and covers defects larger than previously described in the literature.

## Background

Various techniques to reconstruct complex chest wall defects have been described, among them—however not considered often—is the EOM flap [1–3]. It has both a single dominant blood supply and associated multiple segmental vascular pedicles of the deep circumflex iliac artery. It is most often used as a pedicled flap and is straight forward in harvest and inset. The muscle has been described in reconstructing defects to the level of the 2nd intercostal space and well past the thoracic midline [2, 4]. Its large surface area and ability to rotate both caudad and cephalad make this flap ideal in reconstructing thoracic, abdominal, and groin defects of various sizes. When used in chest reconstruction, the flap’s superior margin coincides with the defects caudal border, and as such, no patient repositioning is required during harvest and or inset. The flap can be harvested with or without the anterior abdominal fascia, which when included does provide support to the chest reconstruction. However, this mandates that the resultant fascial defect be reconstructed with mesh or plication-imbrication of the internal oblique fascial sheath [1, 2]. We report on a patient with a chest wall soft tissue defect measuring 900 cm^2^ (30 cm × 30 cm) after angiosarcoma resection, in whom an ipsilateral pedicled EOM flap was used for reconstruction of a large chest wall defect, which was re-elevated 1 year later for an aortic valve replacement.

## Case presentation

**Fig. 1:**
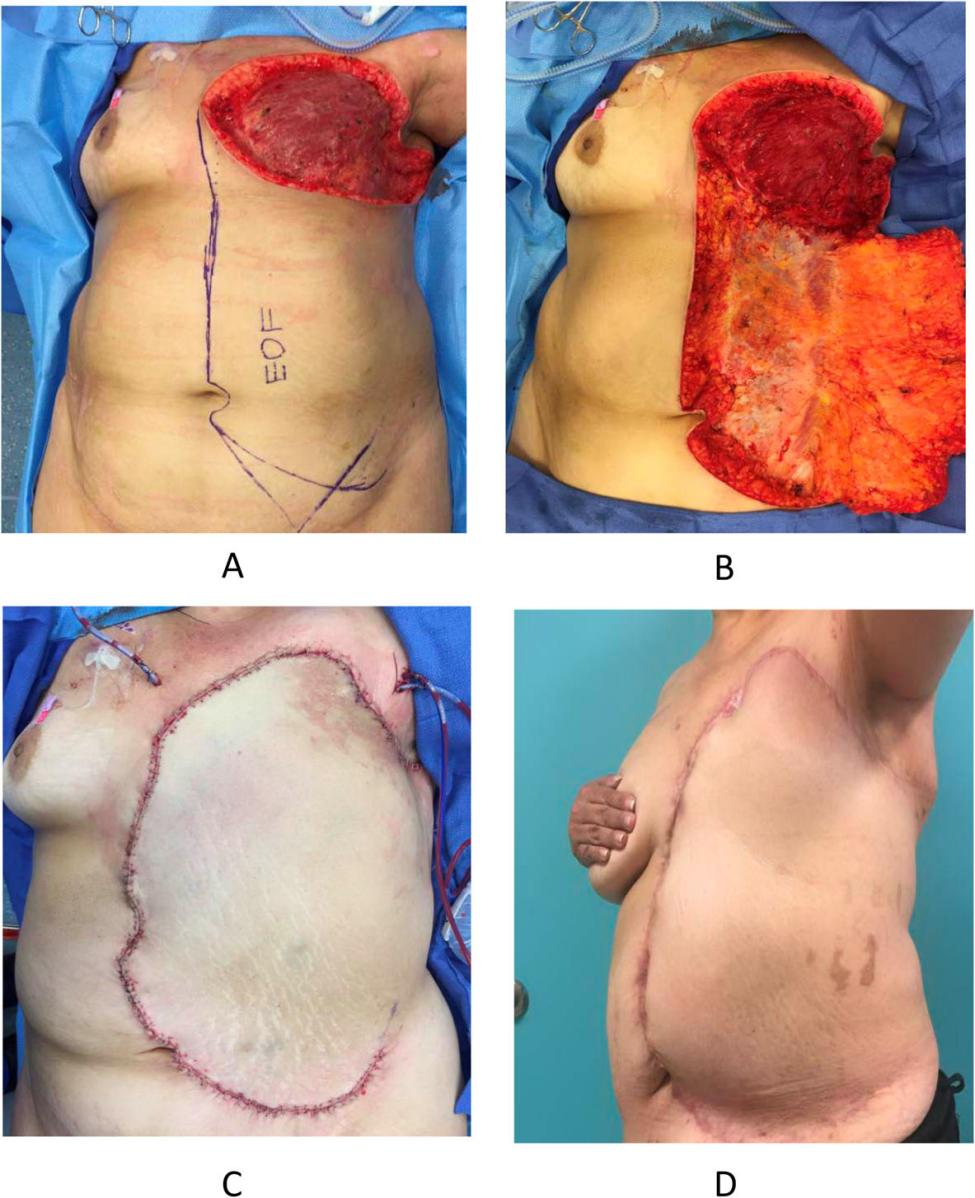
Intraoperative thoracic wall defect status post wide local excision. **a** Resected tumor requiring external oblique musculocutaneous flap. **b** Image showing exposed external oblique muscle used for repair. **c** Postoperative results showing a repaired defect of 30 cm × 30 cm. **d** Image showing appropriate wound healing after 6 months status post procedure

**Fig. 2:**
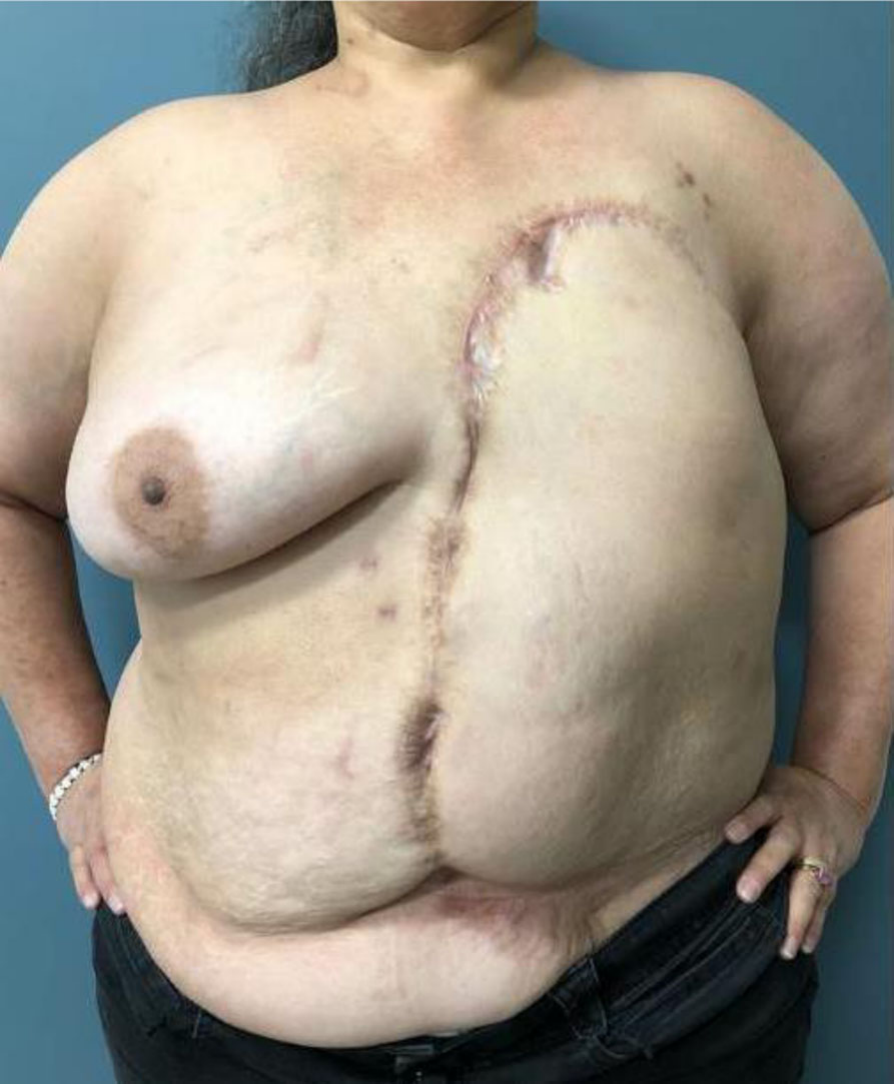
Postoperative thoracic wall defect status post EOM flap. Image illustrates postoperative results status post re-elevation and mobilization of the left EOM flap required for replacement of the aortic valve. The EOM flap has healed well even with elevation and placement

The EOM flap was performed in a 57-year-old female for chest wall reconstruction after resection of a grade 3 angiosarcoma of the left breast. The patient first underwent a modified radical mastectomy without breast reconstruction, followed by wide excision of the chest wall skin once angiosarcoma was confirmed. The EOM flap was used for delayed reconstruction of the patient’s chest wall defect which measured approximately 30 cm × 30 cm (Fig. 1), a surface area of 900 cm^2^. The flap was elevated without the anterior rectus sheath, in the inter-fascial planes between the deep surface of the external oblique muscle and the superficial surface of the internal oblique, with minimal muscle mobilization, and with no sacrifice to the vascular pedicle or perforating vessels from the external oblique to the subcutaneous flap, which were all clearly identified and preserved during the procedure for safety precautions. It was then inserted with minimal tension. Further mobilization could easily have been performed with complete elevation of the muscle to the posterior axillary line and detachment of the EOM off its costal origins and its iliac crest insertion; care taken to avoid injury to its blood supply. The patient was discharged postoperative day #7 and was directed to shower and perform local wound care with Xeroform and abdominal pad dressings. The patient was followed in the office every 4–6 weeks for 6 months. Serial physical exams revealed some epidermolysis and partial thickness necrosis that was appreciated at the superior medial borders, measuring collectively less than 4 cm × 2 cm. The patient was instructed to continue with local wound care as described. The patient did not experience any frank wound dehiscence or any other concerning complications. Thirteen months after the initial procedure, the patient underwent replacement of the aortic valve. In anticipation of cardiac exploration, re-elevation, and mobilization of the prior EOM flap was required. Incisions were made over the healed EOM flap extending over the sternum inferiorly allowing for re-elevation and closure without tension. By postoperative day 2, there was a possible area of partial versus full thickness necrosis versus intermittent venous congestion. Local wound care was initiated, and patient was followed throughout her hospital stay and seen as an outpatient every 2 weeks for 2 months postoperatively. The flap remained viable and healed without evidence of frank dehiscence or necrosis (Fig. 2).

## Conclusion

There are multiple techniques in reconstructing complex chest wall defects resulting from a variety of factors including cancer. One technique may be more appropriate than another depending on multiple factors including patient selection and indications. Workhorse flaps used to reconstruct the thoracic wall include the use of the omentum or pectoralis major, latissimus dorsi, or rectus abdominis muscle flaps. Musculocutaneous and or fasciocutaneous flaps have a layer of the skin and subcutaneous tissue that can fill large defects and provide durable skin coverage, with larger flaps affording larger surface areas of the skin. The EOM is a type V muscle based on the Mathes Nahai classification, having multiple small segmental vascular pedicles as well as one major vascular pedicle supplying blood [2, 5]. The dominant vascular pedicle is supplied most often by the deep circumflex iliac artery, a branch of the external iliac artery [6, 7]. Segmental arteries derived from the 5th–12th posterior intercostal arteries form the minor segmental vascular supply to the external oblique. This dual blood supply contributes to the flaps’ dependability as noted by the viability of the flap even after re-elevation, 13 months after the original procedure. The muscle has been described in literature as having the ability to extend up to defects below the 2nd-4th ribs [3, 5] and to cover defects averaging less than 500 cm^2^ [4]. The external oblique is also the largest flat abdominal muscle, but as reported here, can actually cover large surface areas approximating 900 cm^2^ [8]. However, it is important to note several limitations of the EOM flap that have previously been identified. Given the orientation of the external oblique, it is easier for the plastic surgeon to rotate the flap cephalad, making it more ideal for reconstruction of upper chest wall defects given this limited arc. Necrosis is relatively common given the segmental arteries arise from the deep surface of the flap making it more prone to unforeseen vascular damage. Lastly, care must be taken to avoid damage to intercostal nerves during flap elevation, as such nerves reside in the field of operation. However, when other techniques are not available for reconstruction, one can readily turn to the EOM flap [9]. Thus, it is important for a plastic surgeon to have multiple tools in their reconstructive armamentarium. The EOM pedicle flap is one of those tools, not commonly considered in chest wall reconstruction. We report the successful closure of a high thoracic wall soft tissue defect measuring 900 cm^2^ in a patient requiring local wide excision for a grade 3 angiosarcoma of the breast along with re-elevation and mobilization of the EOM pedicle flap for thoracic exploration for an unrelated cardiac procedure. We, as many authors before us, agree that the EOM flap is a reliable, easily harvested flap with great versatility in providing stable durable coverage of larger defects of the chest than previously believed.

## Data Availability

Not applicable.

## Abbreviation

EOM: External oblique myocutaneous flap
